# Ecology of aspergillosis: insights into the pathogenic potency of *Aspergillus fumigatus* and some other *Aspergillus* species

**DOI:** 10.1111/1751-7915.12367

**Published:** 2016-06-07

**Authors:** Caroline Paulussen, John E. Hallsworth, Sergio Álvarez‐Pérez, William C. Nierman, Philip G. Hamill, David Blain, Hans Rediers, Bart Lievens

**Affiliations:** ^1^ Laboratory for Process Microbial Ecology and Bioinspirational Management (PME&BIM)Department of Microbial and Molecular Systems (M2S)KU LeuvenCampus De NayerSint‐Katelijne‐WaverB‐2860Belgium; ^2^ Institute for Global Food SecuritySchool of Biological SciencesMedical Biology CentreQueen's University BelfastBelfastBT9 7BLUK; ^3^ Faculty of Veterinary MedicineDepartment of Animal HealthUniversidad Complutense de MadridMadridE‐28040Spain; ^4^ Infectious Diseases ProgramJ. Craig Venter InstituteLa JollaCAUSA

## Abstract

Fungi of the genus *Aspergillus* are widespread in the environment. Some *Aspergillus* species, most commonly *Aspergillus fumigatus*, may lead to a variety of allergic reactions and life‐threatening systemic infections in humans. Invasive aspergillosis occurs primarily in patients with severe immunodeficiency, and has dramatically increased in recent years. There are several factors at play that contribute to aspergillosis, including both fungus and host‐related factors such as strain virulence and host pulmonary structure/immune status, respectively. The environmental tenacity of *Aspergilllus*, its dominance in diverse microbial communities/habitats, and its ability to navigate the ecophysiological and biophysical challenges of host infection are attributable, in large part, to a robust stress‐tolerance biology and exceptional capacity to generate cell‐available energy. Aspects of its stress metabolism, ecology, interactions with diverse animal hosts, clinical presentations and treatment regimens have been well‐studied over the past years. Here, we synthesize these findings in relation to the way in which some *Aspergillus* species have become successful opportunistic pathogens of human‐ and other animal hosts. We focus on the biophysical capabilities of *Aspergillus* pathogens, key aspects of their ecophysiology and the flexibility to undergo a sexual cycle or form cryptic species. Additionally, recent advances in diagnosis of the disease are discussed as well as implications in relation to questions that have yet to be resolved.

## Abstract 2

Some *Aspergillus* species, most commonly *Aspergillus fumigatus*, can induce allergic reactions and life‐threatening systemic infections in humans. Here, we discuss the potency of *Aspergillus* as an opportunistic pathogen of humans and other animal hosts. We focus on the biophysical capabilities of pathogenic aspergilli; key aspects of their ecophysiology; and their ability to undergo a sexual cycle or form cryptic species. Recent advances in diagnosis of the disease are discussed, as well as outstanding scientific questions.

## Introduction

*Aspergillus* species are widespread in the environment, growing on plants, decaying organic matter, and in soils, air/bioaerosols, in/on animal systems and in freshwater and marine habitats. Aspergilli are also found in indoor environments (surfaces of buildings, air, household appliances, etc.) and in drinking water and dust. The diverse species which make up the *Aspergillus* genus are able to utilize a wide variety of organic substrates and adapt well to a broad range of environmental conditions (Cray *et al*., 2013a). They produce asexual conidia that readily become airborne and are highly stress tolerant, and can produce environmentally persistent sexual ascospores (Stevenson *et al*., 2015a; Wyatt *et al*., 2015a). Although there are several hundred species in the *Aspergillus* genus, there are only a few species which have considerable impacts on human or animal health. Infections are typically caused by *Aspergillus flavus*,* Aspergillus fumigatus*,* Aspergillus nidulans*,* Aspergillus niger* and *Aspergillus terreus*, among other species (Baddley *et al*., 2001; Perfect *et al*., 2001; Enoch *et al*., 2006; Gupta *et al*., in press), with *A. fumigatus* being responsible for more than 90% of infections, followed in frequency by *A. flavus* and *A. niger* (Lass‐Flörl *et al*., 2005; Balajee *et al*., 2009a,b). However, the actual contribution of different *Aspergillus* species in causing aspergillosis varies from country to country and depends on the patient population under study (for some examples, see Table S1 and references therein, supporting information). Furthermore, some infections attributed to the major aspergilli (i.e. *A. fumigatus*,* A. flavus*, etc.) might be actually caused by cryptic species^1^ (see below). Conidia of pathogenic *Aspergillus* strains that are inhaled by humans or animals are usually eliminated by the innate immune system neutrophils and macrophages in immunocompetent individuals. However, depending on the virulence of the fungal strain, immunological status, and/or the host's pulmonary structure and function, *Aspergillus* can lead to a variety of allergic reactions and infectious diseases in immunocompromised individuals. This may progress to invasive and lethal infection of the respiratory system (and/or other tissues), often followed by dissemination to other organs, a condition known as invasive aspergillosis. A locally invasive version of the disease, chronic necrotizing pulmonary aspergillosis, is mainly observed in humans with mild immunodeficiency or with a chronic lung disease. Non‐invasive forms of *Aspergillus*‐induced lung disease include aspergilloma and allergic bronchopulmonary aspergillosis (ABPA) (Kosmidis and Denning, 2015a,b).

Various factors, including facets of modern living, that contribute to increasing numbers of immunocompromised people include: increases in population longevity; environmental pollution; alcoholism; HIV and other diseases; unhealthy levels of personal hygiene; sedentary lifestyles; obesity; modern medical interventions resulting in high rates of use of prosthetic devices in invasive surgery; chemotherapy and radiotherapy in cancer therapy; and solid organ and bone marrow transplantation requiring the clinical use of immunosuppressive drugs (Maschmeyer *et al*., 2007). As a result, the number of research studies investigating aspergillosis is increasing; there were 13 456 peer‐reviewed reports on aspergillosis for the period 2006–2015, when compared with 8313 for 1996–2005 and 3231 for 1986–1995, according to the Thomson Reuters Web of Science database (accessed 28 April 2016).

The success of members of the *Aspergillus* genus as dominant organisms in diverse habitats is attributable to a combination of interacting factors (Cray *et al*., 2013a) resulting in a global ubiquity which particularly contributes to the impact of *A. fumigatus* as a successful opportunistic pathogen. Morphological characteristics, a remarkable stress‐tolerance biology, an ability to penetrate host defences and colonize/damage the host, exceptional ability to generate cell‐available energy, and other aspects of its ecophysiology collectively contribute to its efficacy as a pathogen. The genomes of various *Aspergillus* species have been sequenced and aspects of their stress metabolism, ecology, and interactions with diverse animal hosts, clinical presentations and treatment regimes are well‐characterized. This said insights from these disparate fields need to be fully synthesized to produce an integrated understanding of *Aspergillus* behaviour and capabilities in the context of its exceptional levels of virulence. This review will focus on several aspects by which *Aspergillus*, especially *A. fumigatus*, has emerged as a ubiquitous opportunistic pathogen which increasingly poses an ominous threat to human health and mortality. More specifically, we explore key aspects of its biophysical capabilities and ecophysiology (Tables 1 and 2), and the flexibility to undergo a sexual cycle or form cryptic species, which contribute to the pathogenic potency of *Aspergillus* species during the development of infection. Further, we discuss recent advances in diagnosis of aspergillosis, and go on to discuss unresolved scientific questions in the context of further work needed in relation to both fundamental and applied aspects of aspergillosis.

**Table 1:** *Aspergillus* ecology within the host system

Behaviour of *Aspergillus*	Clinical implications	Additional notes and seminal studies
Entry into and germination within the host tissue		
Conidia produced by aspergilli in the environment are readily airborne due to their hydrophobicity and small size (Taha *et al*., 2005). Conidia can enter host tissue via wounds, ingestion or (more commonly) inhalation (Oliveira and Caramalho, 2014). The extraordinarily small conidia of *Aspergillus fumigatus* and *Aspergillus terreus* (2–3 μm) allow them to invade the nasal cavity, upper respiratory tract and reach the alveoli, where they bind to surfactant proteins through ligand/receptor recognition (Latgé, 1999; Dagenais and Keller, 2009; Lass‐Flörl, 2012; Oliveira and Caramalho, 2014). The hydrophobic character of the conidial surface is lost, and the epithelial cells endocytose the spore (Kwon‐Chung and Sugui, 2013). Imbibition of water is rapid, the conidium becomes metabolically active within 30 min, followed by germination and then the production of hyphae within 6–8 h (Lamarre *et al*., 2008; Kwon‐Chung and Sugui, 2013; Oliveira and Caramalho, 2014; van Leeuwen *et al*., 2016)	Cilia, with their mucus lining, can act as a barrier which prevents microbial infection of lung tissue. However, pathogenic strains of fungi can penetrate this (Kwon‐Chung and Sugui, 2013). Furthermore, fungal infection can occur more rapidly than the host's immune response, which can take up to 24 h (Cramer *et al*., 2011). The multiple types of damage inflicted by the pathogen to host tissue can become irreparable (even with medical interventions) and can lead to death (Lopes Bezerra and Filler, 2004; Filler and Sheppard, 2006)	The water activity of the mucus lining of the lung is likely to be approximately 0.995 (Persons *et al*., 1987) and even propagules of xerophilic fungi germinate at this high value (Stevenson *et al*., 2015a). *Aspergillus flavus* conidia are the largest and the least aerodynamic of the five species of *Aspergillus* most commonly associated with aspergillosis; they have a diameter of 3.5–4.5 μm (Hedayati *et al*., 2007) so they are more easily trapped and removed by mucocilliary clearance (Binder and Lass‐Flörl, 2013). Tolerance to the various stresses encountered upon entry into and growth within the host system is imperative to successful colonization (Table 2)
Colonization and infection		
Germ tubes within the cytosol of an epithelial cell produce proteases which degrade both the epithelial cell envelope and the wall of adjoining blood vessel(s); furthermore, the germlings exhibit a positive trophism for blood (Lopes Bezerra and Filler, 2004; Filler and Sheppard, 2006). Germ tubes grow into the blood vessel, releasing hyphal segments which are thereby distributed throughout the host. Damage inflicted upon penetration of blood vessels can cause haemorrhaging (Lopes Bezerra and Filler, 2004). Once within the blood stream, hyphae induce expression of thromboplastin which promotes coagulation, thereby causing blood clots (Lopes Bezerra and Filler, 2004; Filler and Sheppard, 2006). Hyphal fragments adhere to endothelial cells, secrete proteases to enter the cytosol of the latter, and continue hyphal growth within the vascular tissue/organs (Lopes Bezerra and Filler, 2004; Filler and Sheppard, 2006). Pathogenic *Aspergillus* strains are able to adapt their metabolism to fluctuating nutrient availability. For instance, such strains can obtain amino acids as a nitrogen substrate via production of hydrolases and proteases (Askew, 2008) (see also ‘Biofilm formation’ below). During germination and colonization, pathogenic aspergilli must respond/adapt to diverse types of stressors, stress parameters, and other challenges including anoxia, nitrogen deprivation, and antifungal metabolites (Table 2)	*Aspergillus fumigatus* spores are negatively charged, aiding in the attachment to surface proteins of epithelial cells (Wasylnka *et al*., 2001). Indeed, the binding efficiency of *A. fumigatus* spores has been implicated in the superiority of this species as a common (if opportunistic) fungal pathogen (Wasylnka *et al*., 2001). When in a resting state, the conidia are not recognized by the dectin‐1‐receptors on macrophages (white blood cells) due to the outer hydrophobin layer of the former which hides their β‐glucan molecules (Oliveira and Caramalho, 2014)	A study of *A. fumigatus* revealed that survival of phagocytosis by macrophages was facilitated by melanin. The macrophage contains the conidium in a vacuole, but is unable to attack the fungal structure because the mammalian ATPases are inhibited by the melanin within the fungal cell wall, thereby preventing the synthesis of phagosomal enzymes (see Thywißen *et al*., 2011). *Aspergillus* can also utilize catalases and oxidases, which protect the fungal cells from reactive oxygen species released in oxidative bursts by phagosomes (Table 2; Missall *et al*., 2004)
Biofilm formation		
Germ tubes and hyphae/mycelium of a pathogenic strain within the host may not exist as an isolated a pure population which is isolated from other microbes (see below). Furthermore, the fungal biomass does not take the form of a simple colony because the hyphae produce extracellular polymeric substances, effectively creating a biofilm (Seidler *et al*., 2008)	A study of echinocadins revealed that *Aspergillus* biofilms are highly resistant to antifungal therapies (Kaur and Singh, 2014); pathogenic aspergilli can also expel antifungal compounds using multidrug efflux pumps. Furthermore, biofilms which develop *in vivo* are more robust (and retain viability for longer) than those produced *in vitro* (Müller *et al*., 2011; Kaur and Singh, 2014). Such factors make the use of antifungal drugs at elevated concentrations imperative to achieving effective treatment of the infection (Kaur and Singh, 2014)	The formation of *Aspergillus* biofilms *in vivo* was demonstrated relatively recently (Seidler *et al*., 2008); extracellular polymeric substances are known to play roles in tolerance to mechanistically diverse stresses (Table 2; Cray *et al*., 2015a)
Competitive ability		
A human‐ or animal host suffering from aspergillosis is likely to have a perturbed microbiome due to a loss of vigour and/or changes in microbial ecology which result from clinical treatment regimes (Lozupone *et al*., 2012; Kolwijck and van de Veerdonk, 2014). Many *Aspergillus* spp. are ecologically vigorous microbes, able to proliferate in perturbed ecosystems/open habitats (Cray *et al*., 2013a; Oren and Hallsworth, 2014). Upon infection, *Aspergillus* can impact the host microbiome, for instance, by inducing the production of antimicrobial peptides (Kolwijck and van de Veerdonk, 2014). In addition, various secondary metabolites produced by *Aspergillus* spp. can inhibit other microbes, cause apoptosis of competitors, or increase the sequestration of nutrients (Losada *et al*., 2009). Conversely, microbes, such as *Candida albicans*, can produce metabolites that cause apoptosis in *Aspergillus* (Losada *et al*., 2009). In turn, the various changes in the human microbiome can potentially render the host more susceptible to disease. Studies of intraspecies variation between plant‐pathogenic aspergilli found that genotypes associated with the broadest range of hydrolytic enzymes and the highest level of aflatoxin were more likely to outcompete other genotypes (Mehl and Cotty, 2013)	Generally, the survival advantage associated with some genotypes which is conferred by high levels of vigour and intraspecific competition favours more pathogenic strains; a phenomenon that has been well studied in relation to plant hosts (Mehl and Cotty, 2013). In the lungs of cystic fibrosis patients, mixed populations of bacteria and fungi can cause exacerbated bouts of sputum production, increases in fungal proliferation, damage to lung tissue, and the risk of allergic bronchopulmonary aspergillosis, a lung‐based form of the disease characterized by inflammation of local tissues and abnormal dilation of the airways (bronchiectasis) (Whittaker Leclair and Hogan, 2010). Treatments for such patients incorporate both antibacterial and antifungal therapies to avoid increases in the bacterial or fungal loads (Whittaker Leclair and Hogan, 2010)	Initial interactions between pathogenic *Aspergillus* spp. and *Pseudomonas aeruginosa* can be synergistic, followed by antagonism upon biofilm formation. This is characterized by the release of diffusible, extracellular molecules that can inhibit hyphal growth (Skov *et al*., 1999; Kaur and Singh, 2014). A study of *Pseudomonas aeruginosa* and *A. fumigatus* interactions revealed that the bacterium was more inhibitory to *A. fumigatus* in a biofilm than when both species were present without a biofilm (Ferreira *et al*., 2015). *In vitro*,* Aspergillus* spp. have been grown in mixed cultures to obtain secondary metabolites with potent antifungal activities which might be useful as drugs (Losada *et al*., 2009). An *in‐vitro* study carried out at 30°C reported that *A. flavus* can outcompete other aspergilli, including *A. fumigatus*,* Aspergillus niger* and *Aspergillus fischeri* (*Neosartorya ficheri*) and demonstrated that *A. fumigatus* and *A. terreus* were ineffective competitors (Losada *et al*., 2009). At 37°C, however, *A. fumigatus* and *A. terreus* were more competitive, inhibiting growth of all other *Aspergillus* species assayed (Losada *et al*., 2009)
Virulence		
Pathogenic *Aspergillus* strains require virulence factors to successfully infect the host. For instance, adhesion factors, such as hydrophobins, allow the binding of the conidia to host epithelial cells (Latgé, 1999; Tomee and Kauffman, 2000). Toxins, such as gliotoxin, can act as immunosuppressants, preventing a host immune response (Latgé, 1999; Sugui *et al*., 2007; Sales‐Campos *et al*., 2013). Subsequent infection of the epithelial cell, therefore, leads to necrosis enabling fungal proliferation and further dissemination of the pathogen (including infection of deep tissue) (Latgé, 1999; Sugui *et al*., 2007; Sales‐Campos *et al*., 2013)	*Aspergillus‐*mediated inhibition of immune response renders the host more susceptible to additional infections (Latgé, 1999; Whittaker Leclair and Hogan, 2010). It is therefore desirable to inhibit gliotoxin production using antifungals which target this activity (Sugui *et al*., 2007; Scharf *et al*., 2012)	A study of *Aspergillus* mutants in gliotoxin synthesis demonstrated that fungal cells unable to produce the toxin could not induce apoptosis of the host cell, and so exhibited reduced virulence (Sugui *et al*., 2007). A key factor which contributes to virulence is a robust tolerance to stresses encountered within the host system (Table 2; Rangel *et al*., 2015a)
Response to clinical treatment regimens		
The types of antifungals used to treat aspergillosis are polyenes, which bind to sterols within the plasma membrane causing leakage of intracellular substrates; and allyamines, echinocandins, and triazoles, which inhibit the synthesis of essential cell‐wall components (Ellis, 2002; Greer, 2003; Chen *et al*., 2011; Vandeputte *et al*., 2012). Some *Aspergillus* strains can remove antifungals via efflux pumps and secrete polymeric substances, thereby reducing contact with antifungal compounds (Seidler *et al*., 2008). Melanin within the *Aspergillus* cell‐wall can bind to antifungals, thereby protecting the cell (Nosanchuk and Casadevall, 2006). Furthermore, some strains use heat shock proteins and/or sterols to reduce entry of antifungals into the plasma membrane (Blum *et al*., 2013; Lamoth *et al*., 2014)	Fungal strains are commonly encountered which resist specific treatment regimes and, in such cases, infections advance even after diagnosis and interventions using antifungals. For strains resistant to antifungals due to their heat shock protein 90 activity, treatment regimes are needed which target the latter (Lamoth *et al*., 2014). Topical treatment of fungal infections has been achieved using photoinactivation strategies (Bornstein *et al*., 2009) and chaotropic antifungals (Cray *et al*., 2014), thereby circumventing various types of resistance to treatment. Such approaches, however, are unsafe and/or inappropriate for treatment of systemic infections	A study of *A. fumigatus* has shown that, at 24 h, germlings are more resistant to voricanazole than those tested 8 h after incubation began; this correlated with temporal variation in levels of expression of genes coding for efflux pumps (Ranjendran *et al*., 2011). To circumvent resistance associated with drug efflux, it is possible to utilize antifungals, such as echinocandins, which cannot be removed by efflux pumps. In addition, prior to administration of antifungals, the patient should be given medication that targets ATPases, depleting the availability of ATP that is otherwise required for effective functioning of efflux pumps (Cannon *et al*., 2009)

**Table 2:** Stress phenotypes and stress metabolism of *Aspergillus* species^a^

Environmental or stress‐parameter	Responses and adaptations	Tolerance limits and biophysical considerations
Temperature		
High temperature and heat shock	Adaptation to high temperature is a polygenetic phenomenon. A study of *Aspergillus fumigatus* revealed changes in 64 proteins, many of these chaperonins, at temperatures exceeding 40°C (Albrecht *et al*., 2010). The heat‐shock response of *A. fumigatus* is highly efficient; the regulation of genes involved in the TCA cycle and production of chaperonins is linked (Do *et al*., 2009). Heat shock protein 90 acts in both protein folding and fungicide resistance in pathogenic aspergilli (Picard, 2002; Albrecht *et al*., 2010; Lamoth *et al*., 2014). The heat‐shock response of *A. fumigatus* is rapid (< 30 min) relative to that of comparator species (~2 h) (Albrecht *et al*., 2010). At high temperatures, aspergilli increase the mean length of lipids in the plasma membrane and synthesize ergosterol, aiding membrane stability (Fritzler *et al*., 2007; Pohl *et al*., 2011). In a study of *Aspergillus terreus*, ergosterol was found to reduce absorption of the antifungal Amphotericin B, thereby confering resistance to the drug (Blum *et al*., 2013)	The upper temperature‐limit for growth of most pathogenic aspergilli is between 40 and 50°C (Schindler *et al*., 1967; Alborch *et al*., 2011; Sharma *et al*., 2014). However, *A. fumigatus* conidia can survive exposure to temperatures of up 70°C (Albrecht *et al*., 2010). *A. terreus* exhibits optimum growth in the range 30–40°C and *Aspergillus niger* in the range 30–35°C (Alborch *et al*., 2011; Sharma *et al*., 2014). Specialized structures (ascospores) of *Aspergillus fischeri* are highly thermtolerant and able to germinate even after a 50‐min heat shock at 85°C (Wyatt *et al*., 2015a). By contrast, the fungal pathogen *Crytococcus neoformans* has an upper temperature for growth of 37–39°C (Lin *et al*., 2006)
Freeze‐thawing	*A. fumigatus*,* A. terreus* and *Aspergillus nidulans* synthesize glycerol as a cryoprotectant through the activation of the high‐osmolarity glycerol response pathway. Cells can be damaged by factors, such as ice crystals, which rupture the plasma membrane and cause the release of the intracellular components into the environment, and/or lead to cellular dehydration. Trehalose minimizes the formation of ice crystals by interposing itself within the hydrogen‐bond network of water within the cell membrane (Jin *et al*., 2005; Teramoto *et al*., 2008; Duran *et al*., 2010; Wong Sak Hoi *et al*., 2012). During thawing, *A. fumigatus*,* A. terreus* and *A. nidulans* utilize trehalose to stabilize cell membranes, both structurally and also by protecting themselves from oxidative damage (Jin *et al*., 2005)	The presence of trehalose and glycerol enables cells to remain viable, even at temperatures as low as −20°C (Wyatt *et al*., 2015a) due, in part, to the reduction in osmotic stress within the cell. In addition, these compatible solutes maintain the integrity of the lipid bilayer, so cellular processes can occur unhindered (Jin *et al*., 2005; Wong Sak Hoi *et al*., 2012)
Solute activities		
Chaotropicity	Compatible solutes, including glycerol and trehalose, can play essential roles in protection of cells against dissolved substances which disorder the macromolecular systems of *Aspergillus* and other fungi (Hallsworth *et al*., 2003a; Bell *et al*., 2013; Alves *et al*., 2015; Cray *et al*., 2015a). This said, chaotropic solutes like ethanol and urea, and many secondary metabolites with antimicrobial activity do not induce compatible‐solute synthesis according to a study of the xerophile *Aspergillus wentii* (Alves *et al*., 2015). Under chaotrope‐induced stress, microbial cells increase production of proteins involved in protein stabilization, energy generation and protein synthesis; undergo modifications of membrane composition; experience oxidative damage as a secondary stress; and upregulate production of enzymes involved in the removal of reactive oxygen species (Hallsworth *et al*., 2003a; Cray *et al*., 2015a)	A recent study of *A. wentii* demonstrated considerable tolerance limits for a range of chaotropic stressors. For instance, *Aspergillus* was able to grow at CaCl_2_ concentrations of up to 1.34 M (equivalent to a chaotropic activity of > 100.0 kJ kg^−1^) and able to tolerate glycerol at a chaotropic activity of approximately 15.0 kJ kg^−1^ and guanidine hydrochloride at a chaotropic activity of approximately 23.0 kJ kg^−1^ (Alves *et al*., 2015)
Osmotic stress	*Aspergillus* spp. synthesize diverse compatible solutes including glycerol, erythritol, arabitol, mannitol, sorbitol, trehalose and proline (Chin *et al*., 2010; Alves *et al*., 2015). Although each of these can reduce intracellular water activity, glycerol is superior in its ability to depress water activity (Alves *et al*., 2015) and is preferentially accummulated under extreme osmotic stress in *Aspergillus* and other fungi (Hallsworth and Magan, 1994; Ma and Li, 2013; Alves *et al*., 2015; Rangel *et al*., 2015a; Winkelströter *et al*., 2015). For xerophillic *Aspergillus* strains, it has been suggested that inability to retain glycerol in the cell determines system failure under hyperosmotic stress (Hocking, 1993). Retention of glycerol requires transporters, such as aquaglyceroporins, that allow bidirectional transport of glycerol and water in response to osmotic gradients (Lui *et al*., 2015). Fungi can import and accumulate compatible solutes from the extracellular environment (Hallsworth and Magan, 1994). At high NaCl concentrations, cell membrane fluidity is decreased (by increasing the proportion of unsaturated fatty acids) and this aids retention of glycerol (Duran *et al*., 2010)	*Aspergillus* strains are amongst the very small number of microbes able to tolerate concentrations of osmotic stressors that correspond to water activity values of less than 0.700 water activity (Williams and Hallsworth, 2009; Stevenson *et al*., 2015a,b)
Water activity	Low water‐activity is frequently, although not necesarily, accompanied by osmotic stress. For instance, water‐activity reduction can result from high concentrations of substances which freely pass through the plasma membrane (e.g. glycerol; Alves *et al*., 2015) or desiccation (see below). In the absence of an extracellular supply of substances which could be used as compatible solutes, synthesis of glycerol and/or other compatible solutes is needed to retain metabolism or survive at low water‐activity or during desiccation–rehydration cycles (see below; Alves *et al*., 2015; Wyatt *et al*., 2015a,b). Further work is needed to understand *Aspergillus* responses to solute‐induced stresses which are independent of osmotic stress (Williams and Hallsworth, 2009; Alves *et al*., 2015; Stevenson *et al*., 2015a,b). Xerophilic species, such as *Aspergillus penicilliodes*, which has been identified in aspergillosis infections, are able to grow in both high‐solute and low‐solute environments (Williams and Hallsworth, 2009; Stevenson *et al*., 2015a)	*A. penicillioides* is capable of mycelial growth and conidial germination on glycerol‐rich substrates down to at least 0.640 water activity, and extrapolations indicate theoretical minima for hyphal growth and germination of 0.632 (Stevenson *et al*., 2015a) and < 0.600 (A. Stevenson and J. E. Hallsworth, unpublished) respectively. *A. fumigatus* and *A. niger* exhibit optimum growth at 0.970 water activity, and *A. terreus* at 0.940; these species have water activity minima for growth of 0.770, 0.820 and 0.780, respectively (Graü *et al*., 2007; Krijgsheld *et al*., 2012). Villena and Gutiérrez‐Correa (2007) report that activities of *A. niger* enzymes (cellulases and xylanases) are considerably lower at 0.942 than at 0.976 (both within and outside the cell). In addition, transport processes as well as other cellular processes can be inhibited as viscosity and molecular crowding within the cytosol increase (Stevenson *et al*., 2015a,b; Wyatt *et al*., 2015b). During molecular crowding in the cytosol, *in‐silico* modelling indicated that an increased net force is required for diffusion of solutes to take place; in addition, solutes tend to repel each other more strongly (Hall and Hoshino, 2010). The net effect is reduced metabolic activity. Collectively, aspergilli are more tolerant to low water‐activity than are virtually any bacteria or basidomycete fungi ‐ with the exception of some *Wallemia* spp. (Kashangura *et al*., 2006; Stevenson and Hallsworth, 2014; Santos *et al*., 2015; Stevenson *et al*., 2015a)
Hydrophobic stressors	Hydrophobic stressors include hydrocarbons and some secondary metabolites which have antimicrobial activity (Cray *et al*., 2013a,b, 2015a). These stressors (log P > 1.95) preferentially partition into hydrophobic domains of the macromolecular systems, chaotropically disordering them, thereby inducing water stress (Bhaganna *et al*., 2010; McCammick *et al*., 2010; Ball and Hallsworth, 2015). Glycerol and other compatible solutes can mitigate against this activity (Bhaganna *et al*., 2010, 2016; Alves *et al*., 2015; Cray *et al*., 2015a)	*Aspergillus* species are highly tolerant to hydrophobic stressors, including benzene (Bhaganna *et al*., 2010; Cray *et al*., 2013a). Despite some loss of viability, conidia of haploid *A. nidulans* were found to tolerate exposure to saturated benzene fumes (Zucchi *et al*., 2005); *A. niger* can tolerate gaseous hexane up to 150 g m^−3^ (Arriaga *et al*., 2006)
Desiccation‐rehydration		
Longevity	High levels of trehalose and trehalose‐based oligosaccharides facilitate the survival of *Aspergillus* spores during inactivity (Hesseltine and Rogers, 1982; Kwon‐Chung and Sugui, 2013; Wyatt *et al*., 2015b). Studies of *A. niger* conidia reveal that long‐term survival is also associated with an ability to store low amounts of oxygen (20–30 μl mg^−1^ dry weight), allowing for a low level of metabolic activity to maintain viability (Schmit and Brody, 1976; Kilikian and Jurkiewicz, 1997; Jørgensen *et al*., 2011)	Propagules of *Aspergillus* remain viable for periods of decades (20–60 years) and may, indeed, do so for considerably longer periods (Ellis and Roberson, 1968; Hesseltine and Rogers, 1982; Kwon‐Chung and Sugui, 2013)
Rehydration	Trehalose is essential for effective and efficient rehydration as it plays a key role in maintaining membrane structure (Crowe *et al*., 1984). Studies of *A. fumigatus* have also demonstrated a key role of expansin proteins, which increase plasticity of the cell wall during rehydration and cell enlargement, thereby facilitating the osmotic changes which precede germination and ability to invade host tissue (Persons *et al*., 1987; Sharova, 2007; Lamarre *et al*., 2008)	Rehydration and imbibition are extremely rapid (< 30 min); see Table 1
Low pH	H^+^ ATPases make up a large proportion of the *Aspergillus* cell membrane; i.e. approximately 25% of the total number of membrane proteins. A study of *A. fumigatus* showed utilization of H^+^ ATPases to transform the energy from ATP hydrolysis into electrochemical potential, driving the transportation of H^+^ ions (Beyenbach and Wieczorek, 2006). Low pH can irreversibly damage the plasma membrane, including conformational changes to membrane proteins, and cause leakage of ions and metabolites (Mira *et al*., 2010). The plasma membrane acts as an osmotic barrier, such that the cytosol can be maintained at a pH different from that of the environment (Longworthy, 1978). A study of *A. niger* revealed that movement of H^+^ ions across the plasma membrane is rapid, enabling efficient adaptation to pH‐induced stresses, such as those imposed by ammonium metabolism (Jernejc and Legiš, 2004)	*A. niger* has a lower pH limit for growth of 1.5 and *A. fumigatus* is able to grow at pH values as low as 3 (Krijgsheld *et al*., 2012; Kwon‐Chung and Sugui, 2013). In addition, *A. fumigatus*,* A. niger* and *A. terreus* survive optimally under slightly acidic conditions: pH 5.0–6.0 (Krijgsheld *et al*., 2012)
Oxidative stress	*A. fumigatus* is efficient at upregulating production of superoxide dismutase, glutathione peroxidase and catalase, enzymes which detoxify superoxide anions and hydrogen peroxide (Missall *et al*., 2004; Abrashev *et al*., 2005). Without the removal of reactive oxygen species, membrane lipids can be converted to lipid hydroperoxides, by chain reaction, adversely impacting bilayer permeability and integrity. Reactive oxygen species also oxidize thiols, methionines and other amino‐acid residues, thereby impairing protein function (Missall *et al*., 2004). The enzymes involved in oxidative stress response also protect the fungal cell from oxidative bursts produced by phagosomes within the host (Missall *et al*., 2004)	*A. fumigatus* hyphae can tolerate (although are damaged at) ≥ 1 mM hydrogen peroxide (Diamond and Clark, 1982). *A. fumigatus* conidia can tolerate up to 15 mM hydrogen peroxide; at higher concentrations, survival rates are close to zero (Paris *et al*., 2003)
Oxygen availability	The use of aerial hyphae, which enhances oxygen uptake, is a unique adaptation utilized by very few microbes including *Aspergillus* (Steif *et al*., 2014). Some pathogenic aspergilli can function under anoxic conditions. *A. terreus*, for instance, is able to utilize nitrates (via ammonia fermentation) under anoxic conditions and can thereby produce ATP (Steif *et al*., 2014)	Aerial hyphae allow *Aspergillus* to tolerate the low oxygen levels in the lung (as low as 1% partial O_2_ pressure in inflamed tissues) (Lewis *et al*., 1999; Kroll *et al*., 2014). *A. terreus*, for instance, remains active at < 1% partial O_2_ pressure (Kroll *et al*., 2014)
Energy requirements	Exceptional energy‐generating capability has been associated with the record‐breaking stress phenotypes of numerous *Aspergillus* strains (see also Cray *et al*., 2013a). Under NaCl‐induced stress, *A. nidulans* up‐regulates production of glycerol‐6‐phosphate dehydrogenase thereby increasing flux through glycolysis and ATP production (Redkar *et al*., 1998). *A. fumigatus*,* Aspergillus flavus*,* A. niger* and *A. terreus* (and possibly also other aspergilli) possess multiple genes for the same pathways, meaning they are highly efficient at upregulating the TCA cycle, genes involved in metabolism of two‐carbon compounds, pentoses and poyols; giving *Aspergillus* a versatile and efficient metabolism of different carbon sources (Flipphi *et al*., 2009). A study of *Aspergillus oryzae* revealed the production of aerial mycelium which has specialized structures at the ends of the hyphae, with 4.5–5.5 μm diameter pores in their the cell walls (Rahardjo *et al*., 2005a). These structures are characterized by increased oxygen intake and increased rates of respiration (Redkar *et al*., 1998). High concentrations of NaCl stimulate the expression of a gene, *uid*A, which stimulates the glycerol‐6‐phosphate dehydrogenase promotor *gpd*A (Redkar *et al*., 1998). Under chaotrope and NaCl‐induced stresses, *A. niger* is able to produce large amounts of cellulases; equivalent to 10.55 and 10.90 μ ml^−1^ respectively, expediting the breakdown of cellulose that can be used for growth and energy generation (Ja'afaru and Fagade, 2010). When the cellulase and amylase activities of 46 species from 26 fungal genera, including *A. fumigatus*,* A. flavus*,* A. niger* and *A. terreus*, were compared it was found that *A. niger* had the highest amylase activity of these species 1.55 μl 50 mg^−1^ (Saleem and Ebrahim, 2014)	The expression of multiple genes for enzymes that regulate pathways allow fungi to adapt their primary carbon metabolism requirement to the niche they inhabit and confer a selective advantage (Flipphi *et al*., 2009). Cellulose represents a vast reservoir of carbohydrates for saprotropic fungi, and maintaining or upregulating cellulose production under stress typically increases energy generation for fungi in contact with cellulose‐containing substrates (Saleem and Ebrahim, 2014). Regardless of substrate type, energy is essential for multiplication, stress tolerance and competitive ability (Cray *et al*., 2013a)

a *Aspergillus* species are also highly tolerant to low temperatures, alkaline conditions, ionizing radiation, ultraviolet (data not shown), carbon‐ and nitrogen‐substrate starvation (see Table 1 and main text); their tolerance to high ionic strength (Fox‐Powell *et al*., in press) has yet to be established.

## Biophysical capabilities and ecophysiology of pathogenic *Aspergillus* species

Collectively, the aspergilli are remarkable fungi. They are not only environmentally ubiquitous; they are also used as the cell factory of choice for many biotechnological applications (Knuf and Nielsen, 2012). Furthermore, there are numerous aspects of *Aspergillus* cell biology and ecology (including their metabolic dexterity when adapting to nutritional and biophysical challenges) (Tables 1 and 2) which contribute to their status as, arguably, the most potent opportunistic fungal pathogens of mammalian hosts.

Strains of *A. fumigatus*,* A. flavus*,* A. niger* and other *Aspergillus* species can inhabit different types of environments.^2^ These habitats are not only diverse in terms of substrate and implications for fungal lifestyle, but also vary greatly in relation to temperature and water availability regime and the dynamics of other biophysical parameters (Cray *et al*., 2013a; Rummel *et al*., 2014; Lievens *et al*., 2015). Some *Aspergillus* species, including some strains of *A. fumigatus*, are xerotolerant, xerophilic and/or capable of surviving repeated desiccation–rehydration cycles (Williams and Hallsworth, 2009; Krijgsheld *et al*., 2012; Kwon‐Chung and Sugui, 2013; Wyatt *et al*., 2015b), conditions which can promote sporulation. Indeed, *Aspergillus* species are renowned for the large‐scale production of hydrophobic and readily airborne spores, including those which colonize building materials (Ko *et al*., 2002; Afanou *et al*., 2015; Zhang *et al*., 2015). Spores of *Aspergillus* species are among the microbial cells with the greatest longevity; highest tolerances to heat, pressure and chaotropicity; and ability to germinate at the lowest water activity. For example, *Aspergillus* conidia (most commonly implicated in aspergillosis infection) can survive for 60 years or more (Kwon‐Chung and Sugui, 2013); some structures (ascospores) survive exposure to temperatures of 85°C (Wyatt *et al*., 2015a); and their conidia have germinated at 0.640 water activity (and may germinate at < 0.600 water activity according to theoretical determinations; A. Stevenson and J. E. Hallsworth, unpublished), which represents the limit for life on Earth (Stevenson *et al*., 2015a,b).

Entry into the host system is typically via inhalation of, or contact with, *Aspergillus* conidia (Table 1). The small size of *A. fumigatus* conidia (2–3 μm) allow deep penetration of the pulmonary alveoli. Other *Aspergillus* species, such as *A. flavus*, produce larger conidia which can be removed more easily by the mucociliary clearance in the upper respiratory tract (Binder and Lass‐Flörl, 2013). Conidia and other spores are invariably desiccated (Bekker *et al*., 2012; Wyatt *et al*., 2013), and a rapid recovery from desiccation/short lag phase prior to germination is imperative for pathogenic strains to evade immune responses, and successful infection and invasion of host tissue (Kwon‐Chung and Sugui, 2013). An effective host immune response can take up to 24 h in humans (Cramer *et al*., 2011). As a result, *Aspergillus* strains able to penetrate host tissue in a shorter time are more likely to be effective in terms of colonization and subsequent infection of the host. In addition, conidia of *A. fumigatus* and other species contain melanin which can protect against enzymatic lysis, diverse stresses (see below), and can also inactivate the C3 component of the complement system (which usually plays a key role in the clearance of microorganisms) (Jahn *et al*., 1997; Abad *et al*., 2010).

Lung epithelial cells form a monolayer that can often be the initial point of contact between fungus and host (Osherov, 2012). After adhering to the epithelial cells, conidia are rapidly endocytosed by type II pneumocytes (Zhang *et al*., 2005). Subsequent to entry into the epithelial cell, the conidium can germinate, a key aspect here is the adherence and subsequent entry of fungal spores to the lung epithelium (Slavin *et al*., 1988). *Aspergillus* spores form a diffusible product that is able to inhibit the activity of alveolar macrophages and thereby facilitates this process (Nicholson *et al*., 1996). Furthermore, proteases are produced by the germinating spores which can damage the epithelial cells (Kauffman, 2003), and finally, the spores invade the vascular endothelium by passing from the abluminal to the luminal side of the pulmonary endothelial cells (Ben‐Ami *et al*., 2009). This is followed by the emergence of hyphae that can penetrate the abluminal surface of endothelial cells, simultaneously causing cell damage (Table 1). In severely immunocompromised individuals, following angioinvasion, hyphal fragments can disseminate haematogenously leading to invasion of deep organs (Filler and Sheppard, 2006). Some details relating to the mechanical penetration of germ tubes and hyphae into, and mycelial extension within, host tissue have yet to be fully elucidated (Table 1). It is clear, however, that proliferation within the blood vessels adds to the potency of invasive aspergillosis since it leads to tissue necrosis at the foci of infection reducing leucocyte penetration as well as effectiveness of antifungal drugs (Filler and Sheppard, 2006).

The biophysical challenges encountered both within and without the host, robust stress‐tolerance biology of *Aspergillus*, ability to compete effectively against other microbes (see below), and other demands of invading host tissue/dealing with immune responses (Tables 1 and 2) all require considerable levels of cell‐available energy. Disparate studies – based on mycelial morphology, stress metabolism, bioinformatic analysis of genomes and ecology – indicate that *Aspergillus* species indeed have an extraordinary capacity for energy generation (Table 2). Bioinformatic analyses of whole genomes of *A. fumigatus*,* A. flavus*,* A. nidulans*,* A. terreus* and other species have discovered duplications in a number of genes encoding enzymes involved in metabolic flux at the level of primary metabolism and energy generation, such as those involved in the citric acid cycle and glycolysis (Flipphi *et al*., 2009). *Aspergillus* species can also grow via the formation of a floccose mycelium, producing aerial hyphae that are capable of enhanced oxygen absorption and increased rates of respiration; thereby increasing energy generation and tolerance to heat or other stresses (Rahardjo *et al*., 2005a,b). Studies on *A. nidulans*, under NaCl‐induced stress, indicate an upregulation of glyceraldehyde‐3‐phosphate dehydrogenase which diverts the utilization of carbon substrate into glycolysis (away from the formation of excessive glycerol) and thereby increases ATP production during stress (Redkar *et al*., 1998). *Aspergillus* species are able to utilize a wide range of substrates, highly efficient at acquiring such resources, and can store considerable quantities of nutrients within the cell; all traits which contribute to their energy‐generating capacity and competitive ability (Cray *et al*., 2013a). Species of *Aspergillus* are also among the most stress‐tolerant microbes thus far characterized in relation to, for example, low water activity, osmotic stress, resistance to extreme temperatures, longevity, chaotropicity, hydrophobicity and oxidative stress (Table 2) (Hallsworth *et al*., 2003b; Williams and Hallsworth, 2009; Chin *et al*., 2010; Krijgsheld *et al*., 2012; Cray *et al*., 2013a; Kwon‐Chung and Sugui, 2013; Alves *et al*., 2015; Stevenson *et al*., 2015a,b; Wyatt *et al*., 2015a). Furthermore, aspergilli exhibit the highest tolerances towards ionizing radiation and ultraviolet radiation among other microbes (Dadachova and Casadevall, 2008; Singaravelan *et al*., 2008).

*Aspergillus* species have diverse adaptations and responses to cellular stress, in addition to the reinforcement of energy‐generating capacity. These include the deployment of biophysically diverse compatible solutes and functionally diverse protein‐stabilization proteins; hyperaccumulation of melanin in the cell wall; oxidative stress responses; ability to resist high temperatures; the production of extracellular polymeric substances (EPS) and formation of biofilms; and the ability to compete with other microbes (Tables 1 and 2). Although individual responses are detailed below, many of these are polygenetic traits and, furthermore, multiple responses/adaptations to stress act in concert and/or are connected at the levels of gene expression, metabolic regulation, physiology and biophysics.

Some *Aspergillus* strains can synthesize and accumulate glycerol to extraordinarily high concentrations (up to 6–7 M; A. Stevenson and J. E. Hallsworth, unpublished), e.g. for osmotic adjustment (Alves *et al*., 2015), and mannitol and other polyols which also have unique properties as protectants (e.g. see Hallsworth and Magan, 1995; Rangel *et al*., 2015a). Aspergilli also produce other amino‐acid compatible solutes which, like compatible solutes, can be effective protectants against chaotrope‐ and hydrophobe‐induced stresses (Bhaganna *et al*., 2010; Alves *et al*., 2015); and produce high levels of trehalose and trehalose‐containing oligosaccharides known to protect against desiccation and rehydration events and temperature changes, especially those which occur upon spore germination (Wyatt *et al*., 2015a,b). *Aspergillus* species are metabolically wired to deploy each of these substances (or a combination of compatible solutes) according to the biophysical challenges, and this versatility has been associated with germination and hyphal growth at water activities which represent the limit for life (see above) and with extreme temperature tolerances; an ability to function at subzero temperature (due to preferential accumulation of chaotropic compatible solutes such as glycerol: Chin *et al*., 2010); and ability to stabilize macromolecular systems under conditions which can disorder membranes and other macromolecules (see Ball and Hallsworth, 2015 and references therein)^3^ ; and a high level of competitiveness (Cray *et al*., 2013a). Rehydrating and germinating spores within human or other hosts are subject to biophysically violent changes in hydration, water activity and osmotic stress. Furthermore, cells can undergo temperature changes and may be exposed to chaotropic or hydrophobic substances, such as breakdown products of insect cuticles (Gao *et al*., 2011; Cray *et al*., 2015a). In addition, antimicrobials produced by microbes or the animal host commonly‐like many environmental substances ‐ exhibit the same mode‐of‐action (Fang, 1997; James *et al*., 2003; Hallsworth *et al*., 2007; Cray *et al*., 2013b,b, 2015a; Pedrini *et al*., 2015; da Silva *et al*., 2015; Yakimov *et al*., 2015; Bhaganna *et al*., 2016). Such substances can modify the outcomes of interactions between diverse cells types, although this may reduce or promote infection, depending on a variety of biotic and abiotic factors (Cray *et al*., 2013a, 2015b, 2016; Suryawanshi *et al*., 2015). The complexity and versatility of the compatible solutes produced by *Aspergillus* are akin to those produced by environmentally ubiquitous, tenacious and competitive bacteria such as *Pseudomonas putida* (Cray *et al*., 2013a). These compatible solutes play key roles in various types of habitat‐relevant stresses for diverse types of pathogenic aspergilli (Tables 1 and 2; Cray *et al*., 2013a; Rangel *et al*., 2015a,b).

The activities of protein stabilization proteins (e.g. heat shock proteins, cold shock proteins and chaperonins) are essential to enable microbial metabolism under extreme conditions, can support competitive ability, and can even expand microbial growth windows in relation to biophysical parameters (Table 2; Ferrer *et al*., 2003; Cray *et al*., 2013a). Such proteins may enhance the flexibility of proteins at low temperature (Fields, 2001; Ferrer *et al*., 2003), and stabilize protein structure at high temperature or under chaotropicity‐mediated stressors induced by chaotropic solutes, hydrophobic stressors and solvents (Table 2; Hallsworth *et al*., 2003a; Bhaganna *et al*., 2010, 2016; Cray *et al*., 2015a). One study on *A. fumigatus* identified changes in 64 proteins at temperatures exceeding 37°C, many of these acting as chaperonins (Albrecht *et al*., 2010). Furthermore, *A. fumigatus* appears to downregulate genes involved in carbohydrate metabolism at high temperatures in a way that is linked to the upregulation of heat shock proteins, thereby enhancing the speed, efficiency and efficacy of heat shock response (Do *et al*., 2009). Studies on *A. nidulans* have identified PalA, a protein, which induces an efficient increase in the production of protein stabilization at extreme pH values (Freitas *et al*., 2011).

Melanin has been quantified in *A. fumigatus*,* A. flavus* and *A. niger* at values of 3.4, 1.4 and 2.2 mg ml^−1^ respectively (Allam and Abd El‐Zaher, 2012; Pal *et al*., 2014). In *Aspergillus* spores (as well as their hyphae), this pigment protects against oxidative stress, ultraviolet radiation, ionizing radiation and high temperature by enhancing the rigidity of the cell wall (Dadachova and Casadevall, 2008; Schmaler‐Ripcke *et al*., 2009; Allam and Abd El‐Zaher, 2012; Upadhyay *et al*., 2013; Ludwig *et al*., 2014). It can act as a barrier to host defences (including the generation of free radicals by host macrophages) as well as being able to bind and thereby neutralize antifungal drugs (Nosanchuk and Casadevall, 2006; Upadhyay *et al*., 2013). Additionally, melanin enables survival of conidia after macrophage phagocytosis, by blocking phagolysosome acidification allowing germination and liberation from the phagocytic cell (Slesiona *et al*., 2012 *)*.

Aspergilli, including some of the species associated with aspergillosis, are highly resistant to mechanistically diverse, cell surface acting inhibitors as well as various heavy metals (Ouedraogo *et al*., 2011; Jarosławiecka and Piotrowska‐Seget, 2014; Luna *et al*., 2015). Heavy metals, chaotropic substances, heat and other stresses can induce lipid peroxidation and an oxidative stress response in microbial cells, including high levels of antioxidant enzymes (Hallsworth *et al*., 2003a; Abrashev *et al*., 2008; Luna *et al*., 2015). In *A. niger*, responses to oxidative stress include increased production of antioxidant enzymes and/or increased concentrations of metabolites with antioxidant activity (Gaetke and Chow, 2003; Luna *et al*., 2015). Production of enzymes, such as superoxide dismutase, catalase, glutathione peroxidase, glutathione S‐transferase and glutathione reductase is increased by up to 25% in response to copper‐induced oxidative stress in *A. niger* (see also Table 2; Luna *et al*., 2015). Such enzymes detoxify superoxide anions and hydrogen peroxide, although in each case the mechanism may differ (Table 2 and references therein).

By comparison with other disease‐causing species, *A. fumigatus* is more thermotolerant and ascospores can survive temperatures of 85°C (Wyatt *et al*., 2015a). Growth is feasible at 55°C and is optimal at 37°C (Beffa *et al*., 1998; Ryckeboer *et al*., 2003). Two genes, *thtA* and *cgrA*, are believed to be involved in the thermotolerance of *A. fumigatus*, but they do not seem to contribute to pathogenicity (Chang *et al*., 2004; Bhabhra and Askew, 2005). Yet, no conserved set of genes has been firmly linked to thermotolerance or fungal growth at different temperatures (Nierman *et al*., 2005). Do *et al*. (2009) suggesting that thermotolerance might be due to the efficient regulation of metabolic genes by heat shock proteins.

Further, it has become clear that *Aspergillus* species can produce biofilms on abiotic or biotic surfaces, an ability which impacts clinical medicine (reviewed in Ramage *et al*., 2011). Previous studies revealed that biofilm formation by *Aspergillus* is induced by a complex interplay of different fungal constituents, such as cell wall components, secondary metabolites and drug transporters (Fanning and Mitchell, 2012). Biofilm formation and production of EPS is an important determinant in the development of aspergillosis (Table 1) as EPS and biofilms can also protect against stresses induced by antimicrobials and microbial competitors (Cray *et al*., 2013a and references therein).

The main classes of antifungas used for treatment of aspergillosis are: inhibitors of the ergosterol biosynthesis pathways (i.e. triazoles and allylamines); compounds which bind to sterols thereby damaging cellular membranes i.e. (polyenes); and compounds which act as inhibitors of synthesis of 1,3‐β‐d‐glucan, an important cell‐wall component (i.e. echinocandins) (Ellis, 2002; Greer, 2003; Chen *et al*., 2011; Vandeputte *et al*., 2012). Susceptibility/resistance of *Aspergillus* strains to antifungals can vary; e.g. some may possess mutations in specific genes, such as the *cyp51* gene encoding a 14‐α‐demethylase involved in the ergosterol biosynthesis pathway (Vermeulen *et al*., 2015), heat shock proteins, melanin (see above), efflux pumps and/or biofilm formation (Seidler *et al*., 2008; Kaur and Singh, 2014; Oliveiria and Caramalho, 2014). EPS can prevent diffusion of echinocadins into the biofilm, thereby protecting the fungus (Seidler *et al*., 2008). *Aspergillus* strains occupy diverse habitats, whether located within a human host, soils or other environments (Delhaes *et al*., 2012; Cray *et al*., 2013a). The genomes of *Aspergillus* species typically have large numbers of clusters of secondary metabolite biosynthetic genes and are capable of producing diverse types of antimicrobial substances (Cray *et al*., 2013a), contributing to their ability to thrive and dominate in diverse microbial communities. For instance, Flewelling *et al*. (2015) found that *A. fumigatus* isolate AF3‐093A produces antimicrobials, such as flavipin, chaetoglobosin A and chaetoglobosin B, which are potent inhibitors of bacteria including *Staphylococcus aureus*, methicillin‐resistant *S. aureus* and *Mycobacterium tuberculosis* H37Ra. *Pseudomonas aeruginosa*, which commonly infects the lungs of cystic fibrosis patients (Smith *et al*., 2015), releases metabolites that are known to inhibit fungal growth (Mowat *et al*., 2010). This bacterium – notorious for its ecologically aggressive character as a microbial weed (Cray *et al*., 2013a) – can, for instance, inhibit biofilm formation by *A. fumigatus*. It does not appear to break down extant *A. fumigatus* biofilms (Mowat *et al*., 2010). Furthermore, *P. aeruginosa* has been found to inhibit formation of mycelium, upon germination of *A. fumigatus* conidia, by approximately 85% relative to mycelial biomass from control *A. fumigatus* conidia that were not exposed to bacterial cells (Mowat *et al*., 2010). Nevertheless, *A. fumigatus* can also be an effective competitor of *P. aeruginosa*, and strains of this fungus have frequently been isolated from the lungs of cystic fibrosis patients (Mowat *et al*., 2010); outcomes of such interspecies interactions are determined by a complex range of interacting variables (Cray *et al*., 2013a; in press). Metabolic versatility of *A. flavus*,* A*. *nidulans* and other *Aspergillus* species has also been associated with ecological vigour in nutritionally diverse environments, including host tissues (Cray *et al*., 2013a; Mehl and Cotty, 2013). The ability of *A. flavus* to produce a broad spectrum of degrading enzymes and to infect a wide variety of plant or animal hosts, and to use non‐living substrates suggests it is an opportunistic pathogen capable of subsisting on a diverse range of nutritional sources (Mellon *et al*., 2007; Mehl and Cotty, 2011). Furthermore, drug‐resistant strains of *A. fumigatus* do not appear to suffer from any reduction in ecological fitness (Valsecchi *et al*., 2015).

## Clinical manifestations and diagnosis of aspergillosis

Although the main portal‐of‐entry and site‐of‐infection for *Aspergillus* in human hosts is the respiratory tract, other foci for penetration and infection have also been described (Lortholary *et al*., 1995; Denning, 1996). The clinical manifestations of aspergillosis vary and can be divided into three main categories, according to the location and extent of colonization and invasion (both of which are influenced by the fungal virulence and immune response of the host); these are (i) allergic reactions, (ii) chronic pulmonary aspergillosis and (iii) invasive aspergillosis. *Aspergillus* species can also colonize the host without causing a systemic infection – at sites such as the eyes, ears and skin – although reports of non‐invasive *Aspergillus* within such body locations are considerably less common than those of aspergillosis (Richardson and Hope, 2003). Allergic diseases caused by *Aspergillus* can be associated with asthma, sinusitis and alveolitis and occur following repeated exposure to conidia and/or *Aspergillus* antigens (Denning *et al*., 2014). In such cases, there is usually no mycelial colonization, so removal of the patient from the environmental source results in clinical improvement (Latgé, 1999). ABPA is considered as an extreme form of *A. fumigatus‐*induced asthma. In this case, the fungus grows saprophytically in the bronchial lumen, resulting in bronchial inflammation (Steinbach, 2008). The conidia trigger an IgE‐mediated allergic inflammatory response, leading to bronchial obstruction (Agarwal *et al*., 2015). Symptoms are recurrent fever, cough, wheezing, pulmonary infiltrates and fibrosis (Barnes and Marr, 2006). ABPA is observed in a small but numerically significant fraction of patients with asthma or cystic fibrosis (1–2% or 8–9% of the total, respectively) (Maturu and Agarwal, 2015).

Chronic pulmonary aspergillosis is a progressive cavitary lung disease, which can be accompanied by development of dense balls of fungal mycelium (that are known as aspergilloma) (Schweer *et al*., 2014). These balls are a non‐invasive, saprophytic form of *Aspergillus* that colonize pre‐existing pulmonary cavities, which were formed during tuberculosis or other pulmonary disease (Steinbach, 2008). People with aspergilloma may be asymptomatic, although many suffer from a persistent and productive cough, haemoptysis and weight loss (Babu and Mitchell, 2015). Regarding the second category, there are different forms of chronic pulmonary aspergillosis, depending on the development of infection and the host's immune status. Most common are chronic necrotizing pulmonary aspergillosis and chronic cavitary pulmonary aspergillosis. Although the first one causes the progressive destruction of lung tissue, chronic cavitary pulmonary aspergillosis can cause multiple cavities, with or without aspergilloma, accompanied by pulmonary and systemic symptoms (Ohba *et al*., 2012).

Finally, a third category of aspergillosis is invasive aspergillosis, representing the most life‐threatening opportunistic fungal infection in patients with reduced immunity. Invasive pulmonary aspergillosis is the most common form of invasive aspergillosis, implying fungal invasion in the lung tissue. Patients at risk are predominantly haematopoietic stem‐cell transplant recipients and patients with haematological malignancies undergoing intensive chemotherapy; however, cases involving non‐neutropenic patients have also been reported (Kosmidis and Denning, 2015a,b). Acute invasive rhinosinusitis is an underdiagnosed form of invasive aspergillosis which most commonly involves the maxillary sinus, followed by the ethmoid, sphenoid and frontal sinuses; this type of infection is aggressive and often fatal (Drakos *et al*., 1993; Middlebrooks *et al*., 2015). Finally, disseminated disease (fungaemia) involves systemic invasion of the brain and other organs, such as kidneys, heart, skin and eyes (Latgé, 1999; Singh and Husain, 2013).

Diagnosis of the different forms of aspergillosis presents a major challenge in medicine for several reasons, including the non‐specific nature of their clinical presentation, the lack of a sensitive and accurate diagnostic assay to ensure an early diagnosis, and the fact that pathogenic aspergilli can only be rarely isolated from infected persons (Thornton, 2010; Lackner and Lass‐Flörl, 2013). The most important diagnostic criteria for invasive aspergillosis are as follows: clinical and radiological evidence of lower respiratory tract infection; biological criteria including direct microscopic evaluation, isolation, culture, and definitive identification of *Aspergillus* from a clinical specimen, or evidence from immunological, serological and/or molecular tests; host‐related characteristics, such as neutropenia or persistent fever in high‐risk patients; and histopathological evidence of infection (De Pauw *et al*., 2008; Paulussen *et al*., 2014). An important advantage of culture‐based assays is that isolates are obtained which can be used in epidemiological studies and for the development of new antifungals that are likely to be effective within clinical treatment regimes. However, an *Aspergillus* strain isolated from an infected patient may or may not be the primary causal agent of the aspergillosis infection as multiple fungal strains, some highly pathogenic and others not, may be present (Álvarez‐Pérez *et al*., 2009; Arvanitis and Mylonakis, 2015; Escribano *et al*., 2015).

A variety of immunological tests are available that can be used to diagnose the disease (Arvanitis and Mylonakis, 2015). Assays based on antibody detection have been successful to diagnose allergic aspergillosis and aspergilloma, while assays for fungal antigen detection showed great potential in diagnosing invasive aspergillosis (Richardson and Hope, 2003; Lackner and Lass‐Flörl, 2013). Further, PCR‐based assays have been developed that can improve early diagnosis of aspergillosis. Advantages of such molecular assays include a high sensitivity, ability to establish diagnosis at the species level and capacity to detect genes that confer antifungal resistance (Segal, 2009). In addition, PCR is fast, inexpensive and can be applied to diverse types of sample, such as blood, sputum and tissue. However, PCR‐based methods have not yet found their place in clinical practice mainly due to lack of standardization (Arvanitis and Mylonakis, 2015). When using PCR, special care must be taken to avoid false‐positive results, e.g. caused by conidia commonly present in the air and airways of non‐infected patients (Bart‐Delabesse *et al*., 1997). The European *Aspergillus* PCR Initiative has made significant progress in developing a standard real‐time quantitative PCR protocol, but its clinical utility has to be established in formal and extensive clinical trials (Gomez, 2014). PCR should, therefore, still be used in conjunction with other methods, such as serological assays or radiological methods, to diagnose aspergillosis (Morrissey *et al*., 2013).

## Sexual cycle and cryptic species: implications for virulence

Recent advances in the fields of genomics, cell biology and population genetics have reshaped our view of how fungal pathogens reproduce and might be evolving. Some species, traditionally regarded as asexual, mitotic and largely clonal, are now being examined in the context of their (cryptic) sexuality (Heitman *et al*., 2014; Varga *et al*., 2014). In this regard, while most known *Aspergillus* species – approximately two‐thirds of the total number have not yet been demonstrated to possess a functioning sexual cycle (Dyer and O'Gorman, 2012), there has been a remarkable discovery of sexual stages (also known as teleomorphs) for aspergilli that were hitherto assumed to be asexual, such as *A. fumigatus* (O'Gorman *et al*., 2009), *A. flavus* (Horn *et al*., 2009b) and *Aspergillus parasiticus* (Horn *et al*., 2009b). Notably, these three species were found to be heterothallic (i.e. with obligate outcrossing), which contrasts with the homothallism (i.e. self‐fertilization) of most sexual aspergilli (Lee *et al*., 2010; Dyer and O'Gorman, 2012). In addition, it is known that some *Aspergillus* species can undergo a parasexual cycle that enables genetic recombination during mitosis (Pontecorvo *et al*., 1953; Lee *et al*., 2010; Varga *et al*., 2014).

The discovery of a sexual cycle for *Aspergillus* species has not been casual, but is the result of years of intense research work and accumulating evidence from different fields, including ‘‐omics’ sciences, population genetics and the analysis of the phylogenetic relationships with sexually reproducing species (Dyer and Paoletti, 2005; Paoletti *et al*., 2005; Álvarez‐Pérez *et al*., 2010a,b; Dyer and O'Gorman, 2012; Heitman *et al*., 2014). Importantly, although the discovery of a functional set of genes necessary for sexual developmental processes (also known as mating type [*MAT*] genes) in the genome sequence of some species is usually given a predominant role in this search for the hidden sexuality of the aspergilli, the diagnostic value of basic mycological techniques, such as paired mating and microscopic observation of the development of mature sexual structures, should not be overlooked.

But, why might an opportunistic pathogen like *A. fumigatus* need to maintain a fully operative sexual cycle when asexual conidia are so abundantly produced and effective as infecting propagules? And how frequently do pathogenic aspergilli reproduce sexually in nature? Unfortunately, the answers to these questions remain unknown and, furthermore, might not be so easy to obtain. A prevailing hypothesis which may lead to an explanation for the maintenance of a sexual cycle in some aspergilli is that sexual reproduction might provide important benefits, such as the possible generation of new combinations of beneficial traits, the purging of deleterious mutations and the formation of thick‐walled fruiting bodies that are resistant to harsh environmental conditions (Lee *et al*., 2010; Dyer and O'Gorman, 2012). Nevertheless, sexual reproduction has, in most cases, a 50% cost (i.e. the sexually reproducing organism is only able to pass on 50% of its genes to a progeny); requires considerable investment in time and energy; and can break apart favourable combinations of alleles, potentially reducing fitness (Lee *et al*., 2010). Despite the inherent advantages of sexual reproduction for living systems, it has been suggested that successful fungal pathogens might be undergoing a slow decline in sexual fertility which, eventually, could lead to permanent asexuality (Dyer and Paoletti, 2005). Upon discovery of the *A. fumigatus* teleomorph, it has been suggested that the sexual fertility of *A. fumigatus* might be limited to some isolates of certain geographically restricted populations (O'Gorman *et al*., 2009). However, sexual fertility has now been demonstrated for many isolates from diverse global locations, and some of these even displayed high mating efficiency (Sugui *et al*., 2011; Camps *et al*., 2012). An apparent decline in sexual fertility has also been suggested for some emerging agents of aspergillosis, such as *Aspergillus udagawae* and *Aspergillus lentulus*, which frequently fail to produce cleistothecia in paired matings or produce ascospores that do not germinate (Sugui *et al*., 2010; Swilaiman *et al*., 2013), but the existence of rare supermater (i.e. highly fertile) individuals within these species cannot be yet excluded.

Another intriguing research question is the possible effects of sexual reproduction on fungal virulence. The main cause of concern for the medical community is the possible emergence of recombinant strains with increased virulence and/or antifungal resistance (Álvarez‐Pérez *et al*., 2010a,b; Heitman *et al*., 2014). In this respect, Camps *et al*. (2012) demonstrated that azole‐resistant isolates of *A. fumigatus* with the TR34/L98H mutation (L98H substitution plus a 34‐bp tandem repeat in the promoter region of the *cyp51A* gene) can successfully mate with azole‐susceptible *A. fumigatus* isolates of different genetic backgrounds and give rise to a recombinant progeny displaying distinct phenotypes. Although a detailed study of the genetic structure of *A. fumigatus* in the Netherlands (where multitriazole resistance first emerged) concluded that the TR34/L98H allele seems to be confined to a single, predominantly non‐recombining population of the fungus (Klaassen *et al*., 2012), sexual reproduction might have played a role in the genetic diversification of azole‐resistant *A. fumigatus* strains (Camps *et al*., 2012).

Mating type related differences in virulence have been explored in some clinically important aspergilli, including *A. fumigatus*. For example, Álvarez‐Pérez *et al*. (2010a,b) found an almost fourfold higher frequency of the *MAT1‐1* than the *MAT1‐2* mating type among *A. fumigatus* isolates obtained from cases of invasive aspergillosis, while both mating types were represented in a similar proportion among isolates of non‐invasive origin. Furthermore, in the same study the authors found a significant association between the *MAT1‐1* mating type and increased elastase activity, which is considered to be a relevant virulence factor (or virulence determinant)^4^ of *A. fumigatus* (Blanco *et al*., 2002; Álvarez‐Pérez *et al*., 2010a,b). The possible association between the *MAT1‐1* mating type and *A. fumigatus* virulence was confirmed in an insect model system (the wax moth *Galleria mellonella*) injected with strains of clinical and environmental origin (Cheema and Christians, 2011). However, as *A. fumigatus* virulence is multifactorial, assessment of the specific contribution of the *MAT* locus in virulence is not possible unless the strains used in the experiments are congenic except for the *MAT* locus. Via the latter approach, Losada *et al*. (2015) have recently demonstrated in three different animal models (mice with chronic granulomatous disease, BALB/c mice immunosuppressed with hydrocortisone acetate and *G. mellonella* larvae) challenged with an isogenic pair of *A. fumigatus* strains of opposite mating types, no difference in virulence between them or in the manner by which these caused the disease. Nevertheless, research experience with other fungal pathogens has shown that differences in virulence between mating types can depend on the genetic background of the strains (see, e.g. Nielsen *et al*., 2005), making necessary the use of different pairs of isogenic strains to reach reliable conclusions. Further research on the role of the *MAT* locus on *A. fumigatus* virulence is therefore required.

The classification of aspergilli has traditionally relied on microscopic and visual determinations of cellular structures and colony morphology as well as key physiological activities (Houbraken *et al*., 2014). However, the use of multilocus phylogenies and comparative genomics has enabled a refinement of *Aspergillus* taxonomy (Houbraken *et al*., 2014; Samson *et al*., 2014). For example, genetic characterizations of isolates hitherto regarded as atypical strains of *A. fumigatus* have resulted in their reclassifications as novel species. These distinct species, that nevertheless share a common morphology, commonly referred to as ‘cryptic’ or ‘*A. fumigatus*‐like’, include *A. lentulus* (Balajee *et al*., 2005) and *Aspergillus felis* (Barrs *et al*., 2013). Cryptic species have also been recognized for *A. niger* (e.g. *Aspergillus awamori*; Perrone *et al*., 2011), *A. parasiticus* (e.g. *Aspergillus novoparasiticus*; Gonçalves *et al*., 2012), *A. terreus* (e.g. *Aspergillus alabamensis*; Balajee *et al*., 2009a) and *Aspergillus ustus* (e.g. *Aspergillus calidoustus*; Varga *et al*., 2008). So far, *Aspergillus* species identification based on molecular biology approaches has typically been based on sequencing of the nuclear ribosomal internal transcribed spacer region (Schoch *et al*., 2012). However, different studies have shown that sequence analysis of some protein‐encoding loci, including the beta‐tubulin (*benA*) and calmodulin (*calM*) genes, provides a superior discriminative resolution (Samson *et al*., 2007, 2014; Houbraken *et al*., 2014). Sequence analysis of the *MAT* loci has also proven useful according to several studies (e.g. Barrs *et al*., 2013; Álvarez‐Pérez *et al*., 2014; Sugui *et al*., 2014). This said, a polyphasic approach which includes morphological, physiological, molecular, biochemical and ecological data is likely to be the most informative for resolving taxonomic differences (Samson *et al*., 2007, 2014).

The prevalence of the cryptic species among pathogenic aspergilli is still unclear, but they could account for > 10% of the total clinical isolates (Balajee *et al*., 2009b; Alastruey‐Izquierdo *et al*., 2012, 2013; Negri *et al*., 2014). Nevertheless, reports on their occurrence vary, which could be due to differences in study design (e.g. selection of patient populations) and/or variations in geographic distribution of some species (Alastruey‐Izquierdo *et al*., 2012). Factors, such as increasing awareness among medical practitioners of the importance of the cryptic species (which in turn may lead to a greater research effort), ongoing development of improved techniques for species‐based identification, may also contribute to discrepancies between frequency reports for cryptic species. Some of the cryptic *Aspergillus* species show a decreased susceptibility to a large number of antifungal drugs when compared with other aspergilli (Alastruey‐Izquierdo *et al*., 2012, 2013; Howard, 2014; Nedel and Pasqualotto, 2014). Therefore, accurate identification of clinical isolates is critical for effective, targeted antifungal treatment (Alastruey‐Izquierdo *et al*., 2012, 2013). Some cryptic species do not have predictable susceptibility patterns and therefore, *in‐vitro* susceptibility testing still remains an invaluable tool to aid directed antifungal therapy (Howard, 2014).

In addition to the increased antifungal resistance generally attributed to the cryptic aspergilli, some studies have reported significant differences in pathogenicity between sibling species. For example, Coelho *et al*. (2011) reported that infection by a fungus first identified as *Aspergillus viridinutans* in an immunocompromised patient led to a distinctive form of invasive aspergillosis characterized by increased chronicity and a propensity to spread across anatomical planes, which contrasts with the rapidly progressive disease which is characterized by a predilection for angioinvasion and haematogenous dissemination typically caused by *A. fumigatus*. Subsequent polyphasic taxonomic re‐examination of one of the isolates from that case (isolate CM 5623) suggested that it belonged to a novel species designated as *A. felis*, which also causes invasive aspergillosis in dogs and cats (Barrs *et al*., 2013). Finally, a recently refined phylogeny placed isolate CM 5623 into a separate clade and justified the proposal of yet another new cryptic representative (designated as *Aspergillus parafelis*) within the broadly circumscribed species *A. viridinutans* (Sugui *et al*., 2014). Notably, *A. parafelis* and the closely related species, *Aspergillus pseudofelis* and *Aspergillus pseudoviridinutans*, which were also proposed as novel taxa in the same study, displayed reduced susceptibility to amphotericin B, itraconazole and voriconazole, and increased virulence in different animal models with respect to the type strain of *A. viridinutans* (Sugui *et al*., 2014). Two implications/consequences of this are: (i) the taxonomy of the genus *Aspergillus* is far from being settled; and (ii) clinicians require some basic knowledge on fungal taxonomy and the cryptic species concept, as these can have consequences for disease management. Furthermore, some cryptic species such as *A. parafelis* and *A. pseudofelis* have shown successful mating under laboratory conditions with related species, including *A. fumigatus* (Sugui *et al*., 2014). In any case, despite these few exceptions of promiscuous mating, interspecies crossings in the section *Fumigati* of genus *Aspergillus* are generally infertile, which suggests that most phylogenetically distinct species are also sexually incompatible (Sugui *et al*., 2014).

### Aspergillus‐related factors implicated in virulence

**Figure 1:**
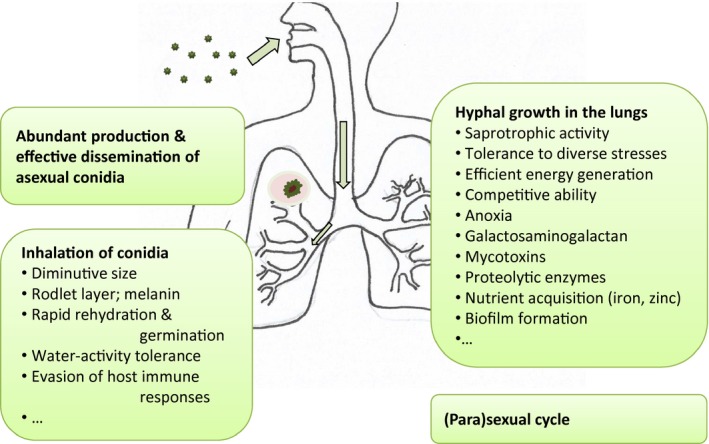
A complex range of *Aspergillus‐* and host‐related factors contribute to the success of *Aspergillus* species as potent pathogens (see also Tables 1 and 2).

Several traits have been postulated to explain the opportunistic behaviour of *Aspergillus*, including fungus‐related factors as well as host‐related factors (see below) (Fig. 1). *A. fumigatus* displays a unique combination of traits that can support its virulence (Dagenais and Keller, 2009). For example, the conidial surface is composed of hydrophobic RodA protein covalently bound to the cell wall, collectively known as the rodlet layer. One important function of this layer is conidial dispersion and soil fixation, but it also masks recognition of conidia by the immune system and hence prevents immune response (Aimanianda *et al*., 2009). Further, recent studies have shown that galactosaminogalactan (GAG), a component of the *Aspergillus* cell wall that is expressed during conidial germination and hyphal growth, has possible anti‐inflammatory effects. GAG induces the anti‐inflammatory cytokine interleukin‐1 receptor antagonist, making individuals more susceptible to aspergillosis (Gresnigt *et al*., 2014). Mycotoxins and fungal enzymes are likely to play an important role in the interaction between *Aspergillus* species and their host. For *A. fumigatus*, several conidial toxins have been described, in addition to a number of toxins released by hyphae (Mitchell *et al*., 1997; Kamei and Watanabe, 2005). Five mycotoxins have been identified in *A. fumigatus*, including gliotoxin, fumagillin, helvolic acid, fumitremorgin A and Asp‐hemolysin. The most studied is gliotoxin, a metabolite in the epipolthio‐dioxopiperazine family that modulates the immune response. Gliotoxin can affect circulating neutrophils, suppresses reactive oxygen species (ROS) production and inhibits phagocytosis of conidia (Scharf *et al*., 2012). Important mycotoxins produced by other *Aspergillus* spp. include aflatoxin, ochratoxin, patulin and citrinin, which can be carcinogenic and/or have a major role in food poisoning (Sweeney and Dobson, 1998). Proteolytic enzymes secreted by *Aspergillus* species, such as serine, metallo and aspartic proteases, are also known to aid virulence (Bergmann *et al*., 2009). *Aspergillus* species secrete a variety of proteases, many of which enable the fungus to saprotrophically utilize animal and vegetable matter. Recently, it has been found that many proteases, e.g. those with elastinolytic activity, also function as virulence factors by degrading the structural barriers of the host and thereby facilitating the invasion of host tissues. Elastin constitutes nearly 30% of lung tissue and elastinolytic activity has been implicated in the pathogenesis of *Aspergillus* (Kothary *et al*., 1984; Blanco *et al*., 2002; Binder and Lass‐Flörl, 2013). Further, trace metal ions, such as iron and zinc, have been shown to contribute to virulence. Iron, for example, is a necessary component of many biosynthetic pathways in fungi and is therefore also essential for pathogenesis. Because free iron is scarce in the human body, *A. fumigatus* produces siderophores (low‐molecular mass iron‐specific chelators) to transport or store ferric ions (Haas, 2012). Zinc is also essential for a wide variety of biochemical processes in fungi, for the adequate regulation of gene expression and thus for cellular growth and development. A clear relationship has been shown between zinc homeostasis and virulence of *A. fumigatus*, which requires the zinc transporters ZrfA, ZrfB and ZrfC for growth within a host (Moreno *et al*., 2007; Amich and Calera, 2014). The wide spectrum of disease states greatly complicates the study of putative virulence factors. Moreover, some virulence factors are active mainly in fungi infecting compromised patients such as those with neutropenia or those receiving, corticosteroid therapy (Hogan *et al*., 1996). It can be expected that additional virulence factors and drug targets can be identified using novel approaches based on whole‐genome sequencing and investigating large collections of fungal strains. In this regard, several studies involving genomic sequencing and subsequent mutant screening have already pointed towards additional gene products that may play key roles in *Aspergillus* pathogenicity (Valiante *et al*., 2015).

### Host‐related factors implicated in virulence

In addition to fungus‐related factors, host‐related characteristics may be equally, or even more, important in development of aspergillosis. Immunity against *Aspergillus* depends on host responses of the innate and adaptive immune system. As described above, *A. fumigatus* is an opportunistic pathogen which is rarely pathogenic in immunocompetent hosts; the immune system kills fungal intruders thereby preventing infection. As such, immunosuppressive therapies and conditions that compromise the immune system trigger the development of aspergillosis (Latgé, 2001). Innate immunity consists of three major lines of defence including anatomical barriers, humoral factors and phagocytic cells (Latgé, 1999). Upon inhalation of *Aspergillus* conidia, the majority of conidia are excluded from the lungs through mucociliary clearance. Lung surfactant enhances agglutination, phagocytosis and killing of conidia by alveolar macrophages and neutrophils. In cystic fibrosis patients, many of these mechanisms are dysfunctional, making these patients highly vulnerable for fungal colonization (Noni *et al*., 2015). Although alveolar macrophages form the first line of defence against inhaled conidia, little is known about their recognition and activation mechanisms. Lectin‐like interactions might be responsible for adherence and uptake of conidia, and also 1,3‐b‐D‐glucan seems to play a role in the conidial binding (Latgé, 2001). The antimicrobial systems via which host cells kill intracellular conidia have not yet been fully characterized.

After fungal germination, polymorphonuclear neutrophils provide the dominant host defence, rendering neutropenic patients at an elevated risk for developing aspergillosis (Kosmidis and Denning, 2015a,b). In the phagocytes, NADPH‐oxidase catalyses the conversion of oxygen to superoxide anion and the generation of ROS displaying antimicrobial activity (Segal, 2009). The ability of neutrophils to attack and kill *Aspergillus* depends on pathogen‐recognition receptors, such as toll‐like receptors (TLR2 and TLR4), dectin‐1, surfactant proteins (A and D) and lectin (Singh and Paterson, 2005; Segal, 2009). Natural killer cells are also important effector cells which play a role in host response to invasive aspergillosis, and are recruited to the lungs as an early defence mechanism (Morrison *et al*., 2003). Natural killer cells are known to mediate immunity against intracellular pathogens (Morrison *et al*., 2003), but their exact role in the immune response against fungi has yet to be studied in detail. Dendritic cells can transport hyphae and conidia of *A. fumigatus* from the airways to the draining lymph nodes and thus initiate disparate responses of T‐helper cells to the fungus (Bozza *et al*., 2002). Following activation of pathogen‐recognition receptors, molecules are released to trigger other players in the immune response to microbial invaders, such as T‐cells, bridging key responders of the innate and adaptive immunity. When the immune system is eventually unable to stop or control hyphal growth, hyphae invade and destroy the surrounding tissue to obtain the necessary nutrients, and, depending on the state of immunosuppression, may cause a disseminated disease (Dagenais and Keller, 2009). Additional research is needed to further unravel the complex interplay between innate and adaptive immunity as key players of aspergillosis, related to different *Aspergillus* strains exhibiting different pathogenicity.

## General conclusions and unanswered questions

Early diagnosis of *Aspergillus* infection has been shown to significantly increase the survival rate of the patient (Nucci *et al*., 2013). However, so far, universally validated diagnostic assays that enable rapid and accurate detection of this potentially deadly fungus have not yet found their way in routine diagnosis of aspergillosis. Furthermore, as aspergillosis can be caused by multiple *Aspergillus* species, including an increasing number of cryptic species, extreme caution should be taken to avoid false negatives (e.g. due to possible unknown differences in the molecular targets of diagnostic tests). Therefore, work is needed on the development of standardized, rapid and highly sensitive diagnostic assays for use in clinical settings without resorting to time‐consuming culturing, e.g. by targeting a conserved gene involved in the pathogenicity of the fungus (Lievens *et al*., 2008). Furthermore, attention should also be given to the occurrence of a sexual or parasexual cycle in *Aspergillus*, as it has been suggested that recombination may give rise to new genotypes with increased virulence (Álvarez‐Pérez *et al*., 2010a,b; Camps *et al*., 2012).

Mortality linked to invasive aspergillosis remains very high despite the availability of new therapeutic strategies. Azole resistance is an emerging problem in *A. fumigatus* and other *Aspergillus* species, and is associated with an increased probability of treatment failure (Denning and Perlin, 2011; Seyedmousavi *et al*., 2014). In addition, particular attention should be given to the increasing occurrence of cryptic species as these are typically linked to an increased antifungal resistance and different pathogenicity (Alastruey‐Izquierdo *et al*., 2012, 2013; Howard, 2014; Nedel and Pasqualotto, 2014). Furthermore, there is a strong appreciation that stress responses and biofilm formation are involved in drug adaptation, which can ultimately lead to development of higher‐level resistance and diminished clinical response (Kaur and Singh, 2014; Perlin *et al*., 2015). In this context, a better understanding of the global magnitude of the azole resistance problem and new therapeutic strategies (e.g. novel dosing mechanisms or introduction of new drugs with novel mechanisms of action, such as biofilm inhibitors) are urgently needed (Denning and Perlin, 2011; Kaur and Singh, 2014).

*Aspergillus* infections pose considerable challenges due to the complexity of the disease, involving pathogen‐, environment‐ and host‐related factors, and the limitations of current diagnostic tools and therapeutic options. Gaining more insight about both the pathogen and host traits as well as the environmental factors, phenotypic traits and evolutionary trajectory which enable *Aspergillus* species to cause disease is crucial to fully understand the interaction between the pathogen and the host, as well as to open new therapeutic perspectives. However, despite intensive research, the inner workings of some of the mechanisms and strategies employed by *Aspergillus* remain enigmatic. For instance, how is it that out of all the fungi, it is *Aspergillus* which is uniquely equipped to evade host defences in such a precise and consistent manner? Why is it that some *Aspergillus* species that are closely related to and share important features with *A. fumigatus*, such as resistance to itraconazole or temperature extremes – e.g. *Aspergillus fischeri* and *Aspergillus oerlinghausenensis* (Houbraken *et al*., 2016) – do not typically behave as opportunistic pathogens. Is it because *A. fumigatus* is more widely spread in the environment, can enter the human host and evade the immune system more successfully, grows well at 37°C or is it better adapted to microenvironments such as the human body that are often characterized by low nutrient and oxygen availability (Tables 1 and 2; Hall and Denning, 1994; Hillmann *et al*., 2015a,b; Kroll *et al*., in press)? *A. fumigatus*, in particular, and aspergilli in general are very highly evolved and successful soil saprophytes and the competition they face in the soil environment has provided some species or strains most probably with the ability to colonize and cause disease in a compromised human or animal host upon entering the lungs (Tekaia and Latgé, 2005). Furthermore, the question arises: what impact these pathogens have had on the structure of the lung and immune system during human and animal evolution? And how can xerotolerant *Aspergillus* species and, moreover, extreme xerophiles, such as *A. niger* and *A. penicillioides*, be so successful as pathogens in the high‐water activity habitat of the human host? Can the energy generation capability of *Aspergillus* play a part in enhancing resistance or tolerance to viral infection and thereby enhance vigour, competitive ability and virulence? It seems paradoxical that a genus which is so ubiquitous in various ecosystems and habitats of the Earth's biosphere is equally competent at invading and proliferating in closed systems, including those represented by food fermentations, microbially contaminated spacecraft (see Rummel *et al*., 2014 and references therein) and an animal or human host. To conclude, only with an integrated research approach bringing together expertise from different disciplines, including mycology, medicine, epidemiology, biopharmaceutical research, ecology, taxonomy and systematics, molecular biology and bioinformatics we will be able to better understand the behaviour and management of this intriguing pathogen.

## Notes

1 Cryptic species are those which can be differentiated using molecular or other analytical techniques and yet are morphologically indistinguishable (Howard *et al*., 2014).

2
*Aspergillus* habitats are detailed in Nieminen *et al*. (2002), Gugnani (2003), Tekaia and Latgé (2005), Womack *et al*. (2010, 2015), Cray *et al*. (2013a,b), Fairs *et al*. (2013), Borin *et al*. (2015) and Hillmann *et al*. (2015a,b).

3 Such conditions include high temperature or heat shock, chaotropicity‐induced stresses and rehydration of dehydrated cells (Crowe *et al*., 1984; Bhaganna *et al*., 2010; McCammick *et al*., 2010; Cray *et al*., 2015a).

4 Virulence factors are molecules produced by pathogens that contribute to the pathogenicity of the organism and enable them to, for example, colonize a niche in the host, evade or inhibit the host's immune response, and obtain nutrients from the host. Pathogens can typically synthesize a wide array of virulence factors.
